# A Histone Deacetylase, Magnaporthe oryzae RPD3, Regulates Reproduction and Pathogenic Development in the Rice Blast Fungus

**DOI:** 10.1128/mBio.02600-21

**Published:** 2021-11-16

**Authors:** Song Hee Lee, Mohamed El-Agamy Farh, Jaejoon Lee, Young Taek Oh, Eunbyeol Cho, Jiyeun Park, Hokyoung Son, Junhyun Jeon

**Affiliations:** a Plant Immunity Research Center, Seoul National University, Seoul, South Korea; b Department of Biotechnology, College of Life and Applied Sciences, Yeungnam University, Gyeongsan, Gyeongbuk, South Korea; c Animal and Plant Research Department, Nakdonggang National Institute of Biological Resources, Sangju, South Korea; d Department of Agricultural Biotechnology, Seoul National University, Seoul, South Korea; Universidad de Córdoba

**Keywords:** histone deacetylation, pathogenic development, RPD3, reproduction, rice blast disease, silencing, rRNA transcription

## Abstract

Acetylation and deacetylation of histones are key epigenetic mechanisms for gene regulation in response to environmental stimuli. RPD3 is a well-conserved class I histone deacetylase (HDAC) that is involved in diverse biological processes. Here, we investigated the roles of the Magnaporthe oryzae *RPD3* (*MoRPD3*) gene, an ortholog of Saccharomyces cerevisiae *Rpd3*, during development and pathogenesis in the model plant-pathogenic fungus Magnaporthe oryzae. We demonstrated that the *MoRPD3* gene is able to functionally complement the yeast *Rpd*3 deletion mutant despite the C-terminal extension of the MoRPD3 protein. MoRPD3 localizes primarily to the nuclei of vegetative hyphae, asexual spores, and invasive hyphae. Deletion of *MoRPD3* appears to be lethal. Depletion of *MoRPD3* transcripts via gene silencing (*MoRPD3^kd^*, where “*kd*” stands for “knockdown”) has opposing effects on asexual and sexual reproduction. Although conidial germination and appressorium formation rates of the mutants were almost comparable to those of the wild type, in-depth analysis revealed that the appressoria of mutants are smaller than those of the wild type. Furthermore, the *MoRPD3^kd^* strain shows a significant reduction in pathogenicity, which can be attributed to the delay in appressorium-mediated penetration and impaired invasive growth. Interestingly, *MoRPD3* does not regulate potassium transporters, as shown for *Rpd3* of S. cerevisiae. However, it functioned in association with the target of rapamycin (TOR) kinase pathway, resulting in the dependency of appressorium formation on hydrophilic surfaces and on TOR’s inhibition by MoRPD3. Taken together, our results uncovered distinct and evolutionarily conserved roles of *MoRPD3* in regulating fungal reproduction, infection-specific development, and virulence.

## INTRODUCTION

Acetylation/deacetylation of histones is an important epigenetic mechanism through which eukaryotic cells regulate the transcription of genes (1, 2). In general, histone acetylation is associated with transcriptional activation, whereas deacetylation has the opposite effect on transcription (3, 4). Histone deacetylases (HDACs) are a group of enzymes that catalyze the detachment of acetyl groups from lysine residues of histones. Some HDACs have been shown to have nonhistone targets, such as transcriptional factors and signaling proteins, and are thus designated lysine deacetylases (KDACs) (5–8). Most HDACs are involved in regulating critical cellular processes, including paired chromatid cohesion, protein conformation, and cellular metabolic processes (9–12). HDACs are classified into four classes, depending on the sequence similarity of proteins and the required cofactors. Class I, II, and IV HDACs require zinc ions, whereas class III HDACs require NAD^+^ as a cofactor for their deacetylase activity (13–15).

*Rpd3* (reduced potassium dependency 3) was initially identified as a suppressor of the growth phenotype under low levels of potassium in a *Trk1* deletion mutant; *Trk1* is a high-affinity potassium transporter in the budding yeast Saccharomyces cerevisiae (16). A follow-up genetic study established *Rpd3* as a global gene regulator in yeast (17). Rpd3 is a class I HDAC that acts as a key component of both small Rpd3 (Rpd3S) and large Rpd3 (Rpd3L) complexes in the yeast (4, 5). To date, the nonhistone targets of RPD3 have not been identified. S. cerevisiae *RPD3* (*ScRPD3*) is known to regulate through its deacetylase activity a functionally diverse group of genes, including those controlling DNA damage (18), responses to extracellular stimuli, such as osmotic and drug stresses (17, 19, 20), and meiosis (17). Among the stress responses, cell growth and proliferation in response to nutrient availability, which is controlled by a conserved regulator of ribosome biogenesis, TOR (target of rapamycin) protein, was shown to be mediated by ScRPD3-dependent changes in chromatin (21, 22). In yeast, hyperactivation of cyclic AMP-dependent protein kinase A (cAMP-PKA) signaling was shown to confer resistance to rapamycin and suppress the repression of ribosome biogenesis imposed by rapamycin treatment, suggesting that TOR and cAMP signaling pathways regulate a number of common functions in parallel (23, 24). For example, it was demonstrated that the PKA and TOR pathways independently control autophagy by regulating the phosphorylation of different sites in Atg13 (25).

Some of the genes orthologous to *ScRPD3* have been studied in filamentous fungi to date. These include *RpdA* in Aspergillus nidulans (26), *HDC2* in *Cochliobolus carbonum* (27), *Rpd3* in Beauveria bassiana (28), and *Hda1* of Ustilago maydis (29, 30). In A. nidulans, it has been demonstrated that *RpdA* is essential for growth and development and that the deletion of this gene is lethal (31, 32). *B.* bassiana, unlike A. nidulans, was viable upon gene deletion, although the mutant showed a drastic reduction in growth, conidiation, and entomopathogenicity and a change in the global acetylome (28). Recently, Botrytis cinerea *RPD3* (*BcRPD3*), Fusarium graminearum *RPD3* (*FgRPD3*), and Magnaporthe oryzae *RPD3* (*MoRPD3*) were studied (33–35). In F. graminearum, deletion of *FgRPD3* was not lethal but resulted in severe growth defects and increased H4 acetylation levels. Overexpression of *BcRPD3* in *B. cinerea* led to defects in growth and pathogenicity. Lastly, overexpression of *MoRPD3* in *M. oryzae* increased asexual reproduction, while rendering the fungus nearly nonpathogenic (35).

In contrast to their yeast counterparts, RPD3 proteins in filamentous fungi have extended C-terminal sequences, in addition to the highly conserved HDAC domain in their N-terminal region. At least in A. nidulans, such C-terminal sequences have been experimentally shown to be important for proper localization of the protein and optimal deacetylase activity (32). For the plant-pathogenic fungi, *Hda1* in Ustilago maydis is the only RPD3 ortholog characterized in detail to date. *Hda1* was shown to regulate teliospore development and act as a suppressor of the biotrophic marker gene *Mig1* (29, 30). The importance of *RPD3* in virulence has also been demonstrated in human-pathogenic fungi, such as Aspergillus fumigatus and Cryptococcus neoformans (36, 37).

Although the functions of *RPD3* have been elucidated in several fungal species, little is known about how RPD3 is implicated in the development and signaling pathways in filamentous fungi. Here, we set out to investigate roles of the *RPD3* gene in the model plant-pathogenic fungus M. oryzae (Pyricularia oryzae). Izawa et al. reported that their attempt to obtain an *MoRPD3* deletion strain was unsuccessful, and thus they did not further characterize the gene (38). To date, *MoHOS2* (HDA One Similar) is the only class I HDAC characterized as a regulator of asexual development and pathogenicity in this fungus (39, 40). *M. oryzae* is a filamentous, heterothallic, hemi-biotrophic fungal pathogen causing the rice blast disease, which is responsible for significant yield loss in cultivated rice (41, 42). The infection process starts when the disseminated conidium lands on the rice leaf surface. Following attachment to the surface, the conidium germinates and differentiates into a specialized infection structure, the appressorium at the tip of the germ tube, upon sensing environmental cues, such as surface hydrophobicity (43). Using turgor pressure generated within the appressorium, the fungus mechanically penetrates the host plant and grows inside plant cells by developing bulbous invasive hyphae that are enclosed in the plasma membrane of the host plant (44). This invasive growth of the fungus eventually leads to the development of disease lesions in the leaves of adult plants and premature death in seedlings (41, 44). Decades of research have revealed that multiple signaling pathways involving cAMP, calcium ions, and mitogen-activated protein (MAP) kinases are involved in surface sensing and the regulation of appressorium morphogenesis (41, 43). A recent study showed that appressorium formation requires inactivated TOR signaling, which mediates cell cycle arrest at the G_2_ phase, and that such TOR signaling and cAMP signaling pathways constitute a feed-forward network, linking the nutrient-sensing and surface-sensing pathways (45).

In this study, we aimed to understand the functions of *MoRPD3* during development and pathogenesis in a model plant-pathogenic fungus, *M. oryzae*, through detailed genetic analyses. Genetic evidence and the localization of the protein indicate that MoRPD3 is a conserved class I HDAC with a C-terminal extension found in other filamentous fungi. Our failure to obtain a gene deletion mutant implies that *MoRPD3* is likely to be an essential gene, the deletion of which renders the fungus inviable. An alternative gene-silencing approach provides evidence that *MoRPD3* is a regulator of asexual/sexual reproduction as well as pathogenic development and that TOR signaling-mediated developmental decisions are incorporated into the regulation of rRNA production by MoRPD3 in *M. oryzae*.

## RESULTS

### Genetic evidence for MoRPD3 as an ortholog of yeast Rpd3.

**FIG 1:**
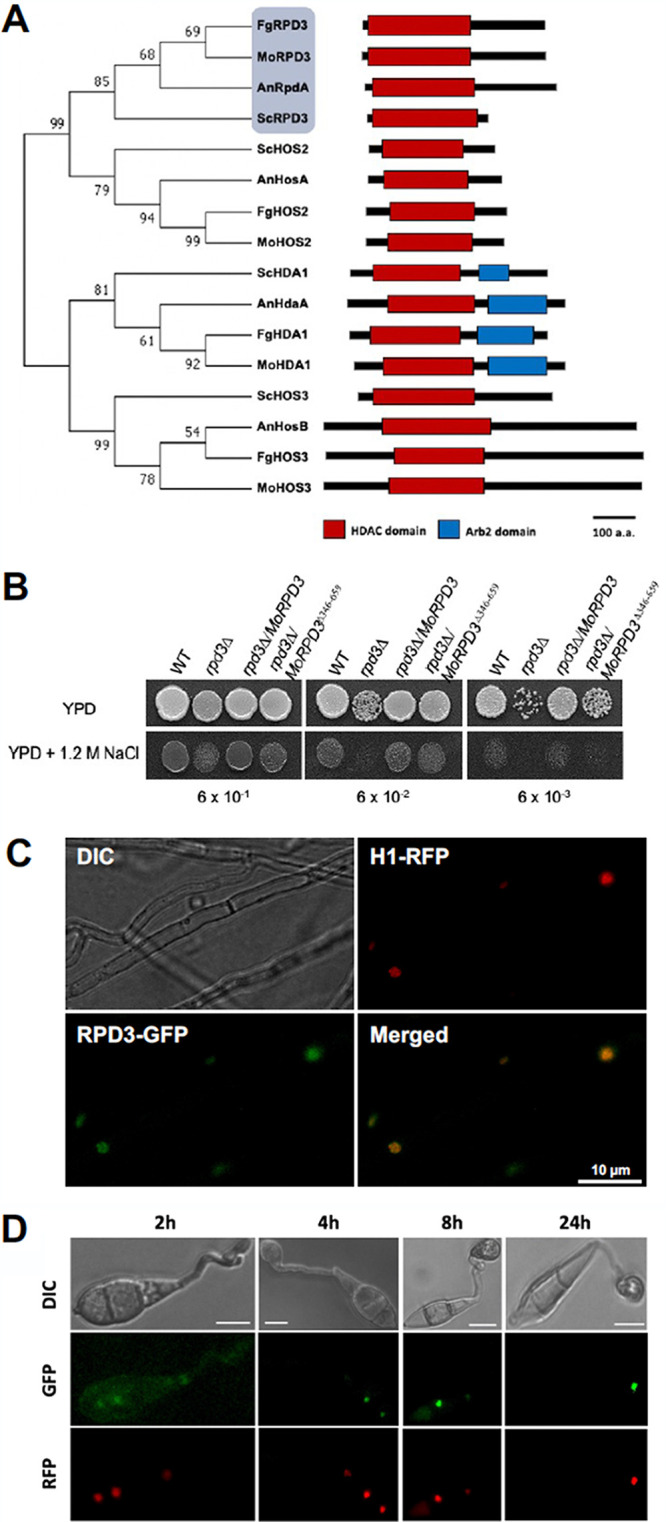
Domain architecture and localization of MoRPD3. (A) The phylogenetic relationship between the MoRPD3 protein and class I HDAC proteins of different fungal species is shown on the left. Bluish gray shading indicates a group that includes MoRPD3. On the right, domain architectures are illustrated. Red and blue boxes indicate histone deacetylase (HDAC) and Arb2 domains, respectively. The phylogenetic tree was constructed using the neighbor-joining method (bootstrap values are indicated at the nodes). a.a., amino acids. (B) Complementation of the Saccharomyces cerevisiae *rpd3*Δ strain by MoRPD3 and MoRPD3-ΔC. Serial dilutions of the wild-type strain (WT; S. cerevisiae ATCC 201389), *Rpd3* deletion strain (*rpd3*Δ; S. cerevisiae ATCC 4011114), and complementation strains (the *Rpd3* deletion strain transformed with *MoRPD3-GFP* or Mo*RPD3*-ΔC-*GFP*) were spotted on YPD plate for 2 days at 30°C with or without 1.2 M NaCl. The localization of MoRPD3-GFP in vegetative hyphae (C) and during conidium germination and appressorium formation (D) is shown. Positions of nuclei are marked with H1-RFP. DIC, differential inference contrast. Scale bar, 10 μm.

Orthologs of ScRPD3, including MoRPD3, were searched and identified using the dbHiMO database, which is dedicated to cataloging curated histone-modifying enzymes from diverse fungal species (http://hme.riceblast.snu.ac.kr) (46). MoRPD3 consists of 659 amino acids and is encoded by a gene (MGG_05857) containing four exons. Phylogenetic analysis showed that MoRPD3, along with other RPD3 orthologs, belong to class I HDACs, including HOS2 (Fig. 1A). Comparison of domain architecture using the InterPro database (47) showed that *MoRPD3* has, in addition to a well-conserved HDAC domain (amino acid positions 20 to 395), an elongated C-terminal region (Fig. 1A). Such a highly extended C-terminal region appeared to be common among RPD3 orthologs in filamentous fungi (see Fig. S1A at https://figshare.com/projects/MoRPD3/124153). The C-terminal motif (amino acid positions 403 to 421), which had been found to be important for the catalytic activity and viability of A. nidulans, was conserved in MoRPD3 as well (Fig. S1B at https://figshare.com/projects/MoRPD3/124153) (28, 31, 32).

In yeast, deletion of the *Rpd3* gene has been shown to cause a reduction in growth. To test whether *MoRPD3* is a functional ortholog of yeast *Rpd3*, we introduced the *MoRPD3*-green fluorescent protein gene (*GFP*) construct into the *Rpd3* deletion mutant of yeast (*rpd3*Δ mutant) and compared the growth of yeast strains. This experiment showed that the *MoRPD3-GFP* construct was capable of complementing the reduced-growth phenotype of the *rpd3*Δ mutant (Fig. 1B). This result clearly demonstrates that MoRPD3 is a functional ortholog of yeast Rpd3 and that the *MoRPD3-GFP* construct produces functional fusion proteins. However, MoRPD3 with a truncated C-terminal region (*MoRPD3^Δ346–659^-GFP*) partially complemented the *rpd3*Δ mutant, suggesting that the C-terminal extension is required for the full functionality or stability of the MoRPD3 protein.

As nonhistone targets of ScRPD3 have not been identified, RPD3 proteins are expected to localize in the fungal nucleus. The PSORT algorithm predicted that *MoRPD3* should be localized to the nucleus (47.8%) and in the cytoplasm as well (26.1%) (48). To test this, we introduced the *MoRPD3*-*GFP* construct (with the native *MoRPD3* promoter) into a strain expressing H1-red fluorescent protein (RFP) as a nuclear marker and monitored GFP signals. In vegetative hyphae, GFP signals were visible in the nuclei (Fig. 1C). In conidia, signals were also detected mainly in nuclei, although faint signals were also found in the cytoplasm (Fig. 1D, leftmost panels). In the germ tube and incipient appressorium, however, no signals were detected (Fig. 1D, leftmost and middle panels). The GFP signal was visible again during appressorium maturation (Fig. 1D, rightmost panels). Taken together, this line of evidence confirms that MoRPD3 is a functional ortholog of the yeast HDAC ScRPD3. It should be noted that in contrast to what occurred with the full-length construct, no GFP signals were observed in strains carrying the *MoRPD3^Δ346–659^-*GFP construct, attesting to the requirement of the C-terminal region.

### Knockdown of the MoRPD3 gene in the rice blast fungus.

**FIG 2:**
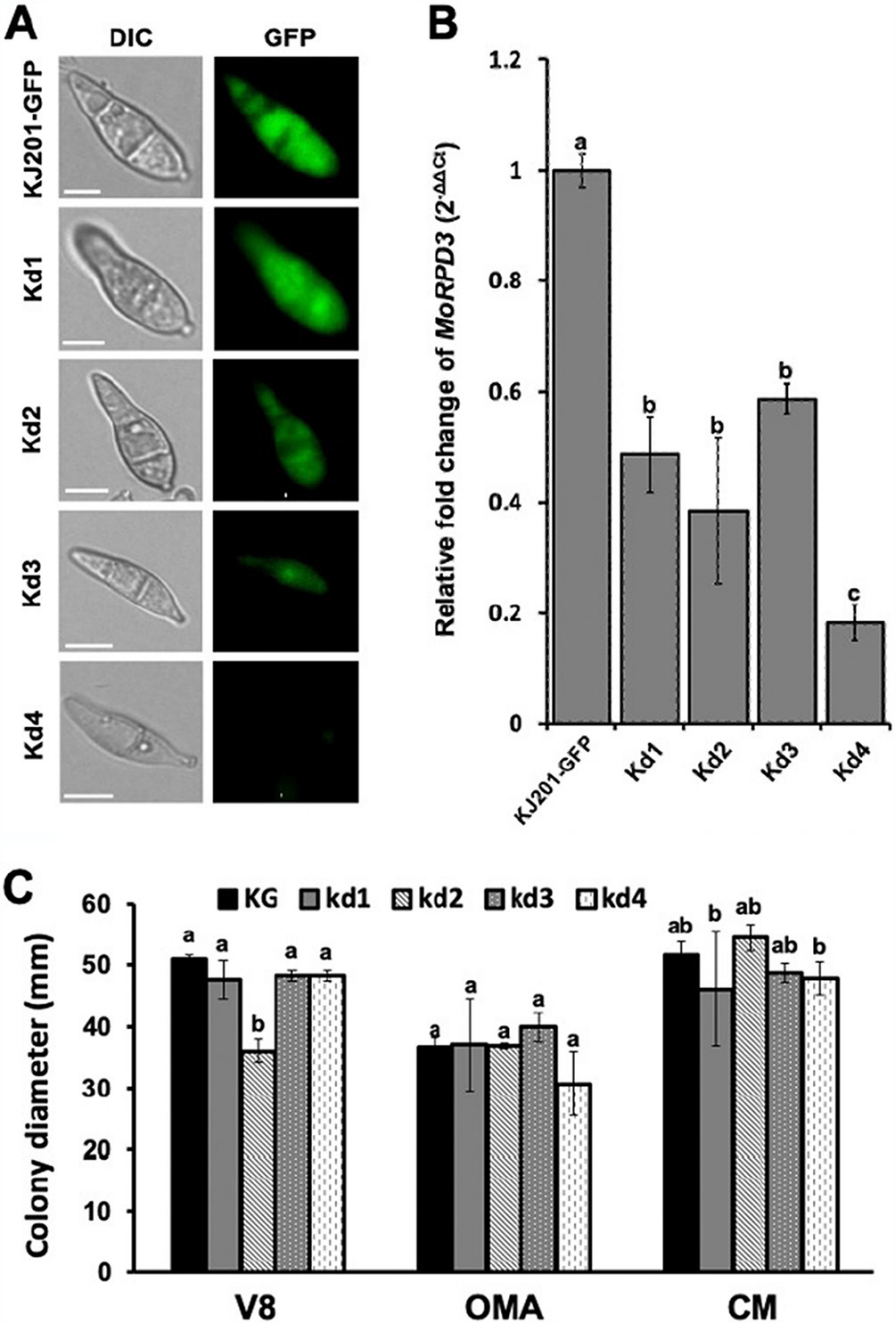
Silencing of the *MoRPD3* gene. (A) Silencing efficiencies of the mutant strains (Kd1 to Kd4) as measured by the reduction in the intensity of the GFP signal under a fluorescence microscope. Scale bar, 10 μm. (B) Silencing efficiencies of the mutant strains as measured in relative transcript abundances of *MoRPD3* using qRT-PCR. (C) Radial growth, which is measured in colony diameter, of silencing mutants on V8, OMA, and CM agar plates, compared to that of the wild-type strain (KG, KJ201 expressing GFP). Error bars indicate standard errors. Different letters on the bars indicate statistically significant differences in mean values (*P < *0.05, least significant difference [LSD] test, *n* = 3).

To investigate the roles of MoRPD3 during development and pathogenesis in the rice blast fungus, we initially attempted to generate a gene knockout mutant of *M. oryzae*. However, we failed to obtain a mutant following PCR-based screening of more than 1,000 transformants in total. This suggests that *MoRPD3* is highly likely an essential gene, abrogation of which renders the fungus inviable. Alternatively, we took a gene-silencing approach using the RNA-silencing vector pSilent-Dual1 (pSD1) (49). In the pSD1 system, the short exon sequences (100 to 200 bp) of the target gene are transcribed as a chimeric RNA with enhanced GFP (eGFP) RNA, enabling efficient screening of the resulting transformants using the reduction in GFP fluorescence intensity as a proxy for gene silencing via the RNA interference (RNAi) pathway. Two rounds of transformation using protoplasts of the KJ201 strain expressing GFP (designated KJ201-GFP or KG), followed by screening based on fluorescence microscopy and quantitative reverse transcription-PCR (qRT-PCR), resulted in four knockdown mutants, which were designated kd1 to kd4. The knockdown mutants exhibited different levels of silencing efficiency, as indicated by fluorescence intensity (Fig. 2A). The qRT-PCR analysis indicated that the transcript abundance of *MoRPD3* in the mutants was reduced by 40% to 80%, compared to that in the wild type (Fig. 2B). When knockdown mutants were grown on V8, oatmeal agar (OMA), or complete medium (CM) agar plates, no significant decrease in radial growth was observed, except for kd2 on V8 medium (Fig. 2C), despite the variation in colony morphology among the mutants (Fig. S2 at https://figshare.com/projects/MoRPD3/124153). Immunoblotting with some of the known substrates for Rpd3 (anti-H3K9ac, H3K18ac, and H4K5ac) showed that the acetylation level of H3K9 and K4K5 increased by 10 to 18% in the silencing mutant, compared to that in the wild type, further supporting the role of MoRPD3 as an HDAC (Fig. S3 at https://figshare.com/projects/MoRPD3/124153).

### Asexual and sexual reproduction in the MoRPD3 knockdown mutants.

**FIG 3:**
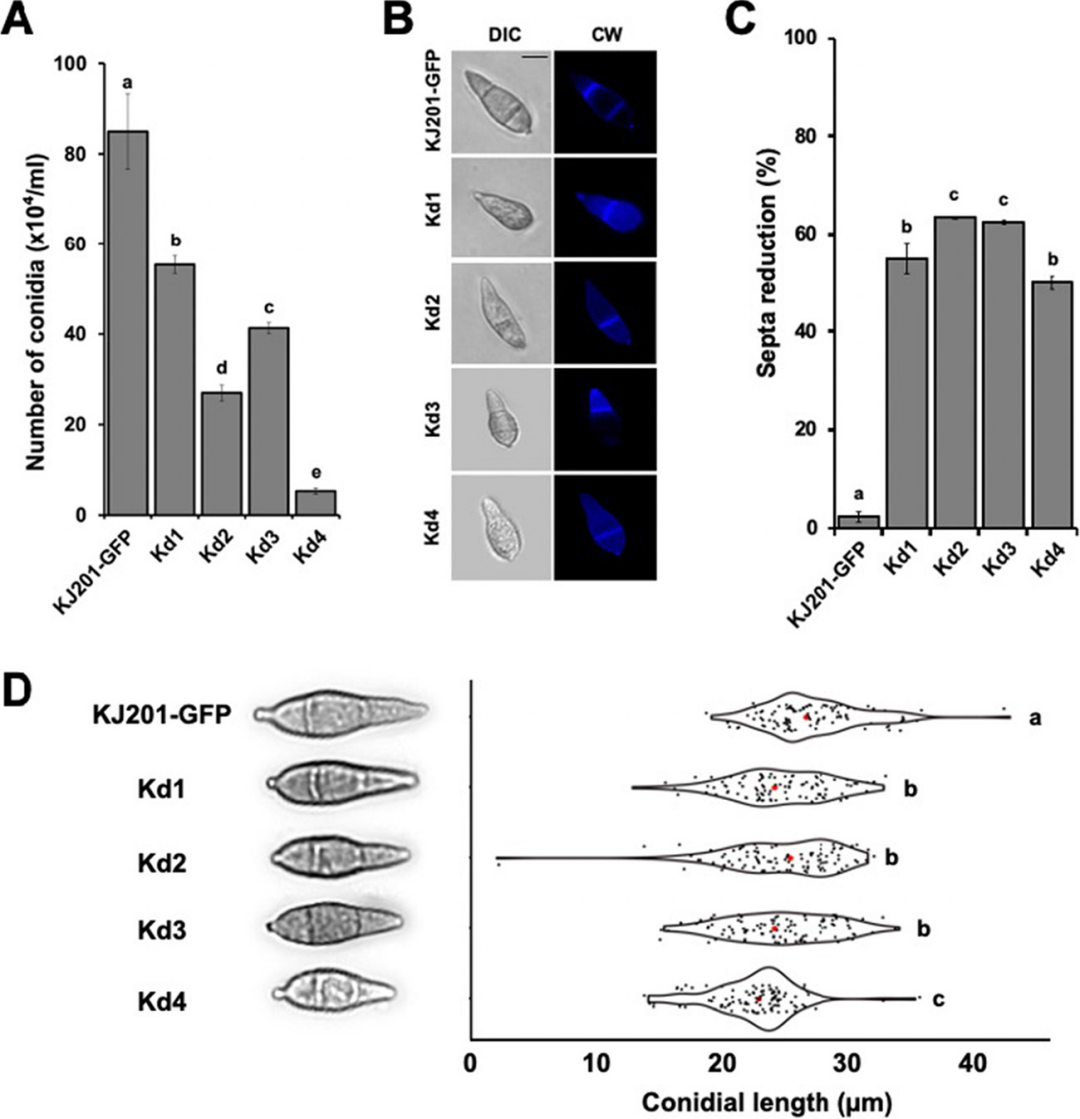
Effect of silencing *MoRPD3* on asexual reproduction. (A) Quantitative analysis of asexual reproduction for silencing mutants (Kd1 to Kd4) in comparison with that of the wild type (KJ201 expressing GFP). Spores were harvested from 9-day-old oatmeal agar plates and enumerated using a hemacytometer. (B) Comparison of conidial morphologies between wild-type and mutant strains. The septa and cell wall were visualized by calcofluor white (CW) staining (right). (C) Proportions of conidia showing a reduction in the number of septa. At least 100 spores were observed for each strain for the reduction in the number of septa. Error bars indicate standard errors. (D) Comparison of spore sizes for silencing mutants and the wild type. Spores showing the typical length of each strain are shown on the left. Red dots within the violin plot indicate mean values. Different letters on the bars indicate statistically significant differences in mean values (*P < *0.05, LSD test, *n* > 100).

When the asexual reproduction of mutant strains was measured as the number of asexual spores produced from the plate cultures, it was shown that conidial production decreased in the mutant strains in a manner that correlated with the degree of *MoRPD3* silencing (Fig. 3A). In addition, close examination of conidial morphology under the microscope revealed that all the mutant strains produced significantly higher proportions (approximately 50% to 64%) of conidia with a single septum than those produced by the wild-type strain with mostly two septa (Fig. 3B and C). The conidia of all the mutants exhibited a length-wise reduction in size, even when they had two septa (Fig. 3D).

**FIG 4:**
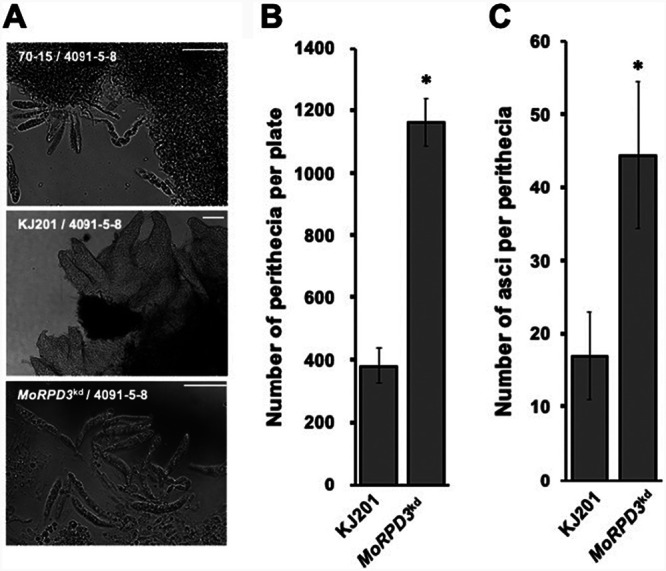
Effect of silencing *MoRPD3* in sexual reproduction. (A) Micrographs showing asci coming out from perithecia following the breakdown of the perithecia with a pipette tip. Scale bar, 10 μm. (B) Comparison of numbers of perithecia between the wild-type and mutant strains. (C) Comparison of numbers of asci between the wild-type and mutant (*MoRPD3^kd^*) strains. Asterisks indicate a statistically significant difference in mean values (*P* < 0.05, *t* test, *n* = 3).

KJ201, which was used as a wild-type strain in this study, is a poorly fertile, female sterile field isolate carrying the MAT1-1 mating locus and thus requires a strain carrying the MAT1-2 mating locus for sexual reproduction. To test whether *MoRPD3* is also involved in sexual reproduction, genetic crosses using a highly fertile 4091-5-8 strain carrying MAT1-2 were undertaken. Laboratory strain 70-15 (MAT1-1), which was used as a positive control, produced a large number of perithecia, each of which contained 40 to 50 asci. As expected, KJ201 produced a much smaller number of perithecia, many of which lacked asci (Fig. 4A to C). In contrast to what occurred with the wild type, the numbers of perithecia and asci produced by the *MoRPD3^kd^* strain were almost comparable to those produced by the 70-15 strain, although female fertility was not restored. This strongly suggests that *MoRPD3* is a negative regulator of sexual reproduction in *M. oryzae*.

### Requirement of MoRPD3 for proper morphogenesis during appressorium formation.

**FIG 5:**
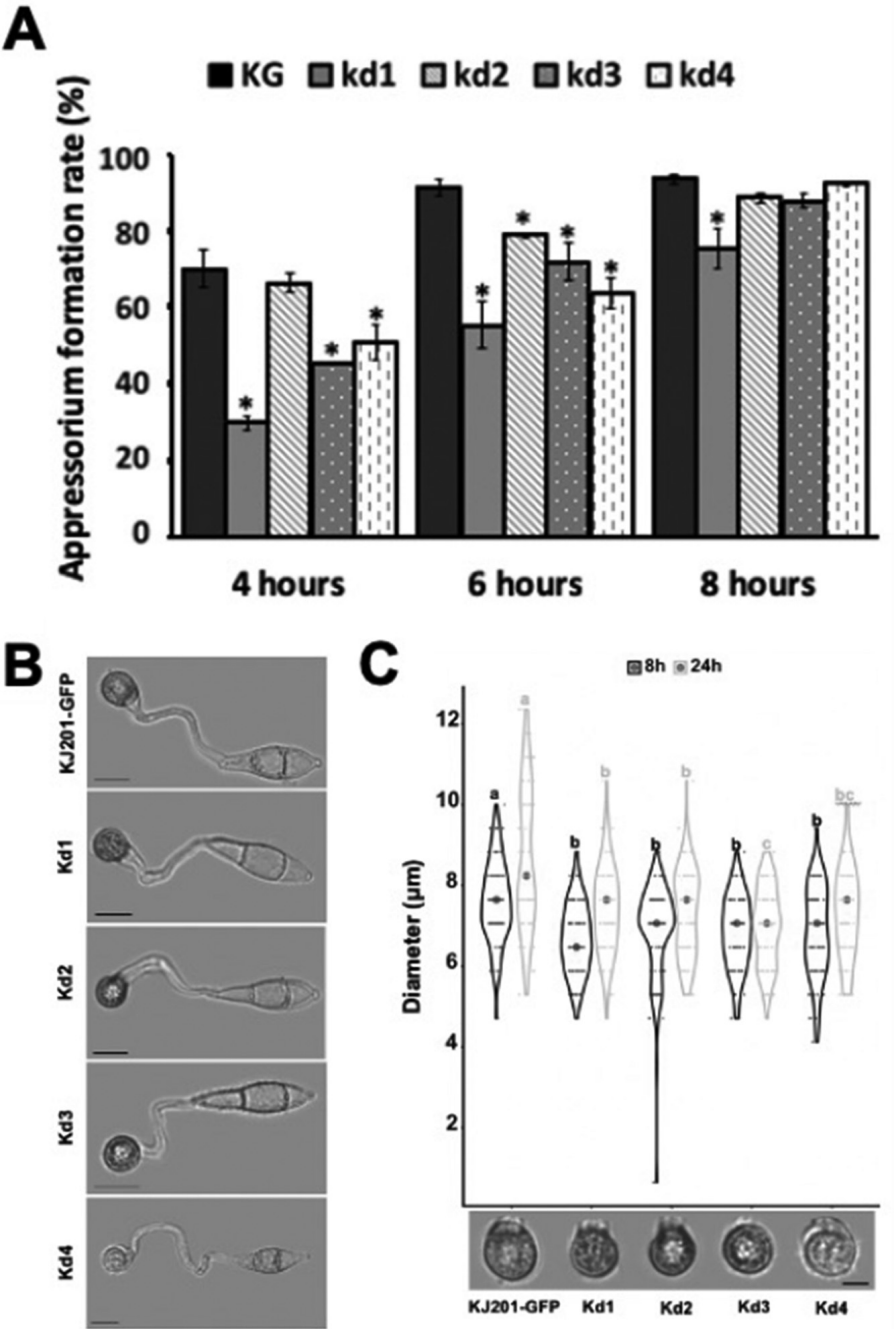
Appressorium formation and morphogenesis in the *MoRPD3* silencing mutants. (A) Appressorium formation rates of the mutants on an inductive (hydrophobic) surface at 4, 6, and 8 h postincubation. Error bars indicate standard errors. Asterisks indicate a statistically significant difference in mean values, compared to values for the wild type (*P < *0.05, LSD test, *n* = 3). (B) Representative images showing size reduction in the appressoria of the mutant strains. Scale bar, 10 μm. (C) Comparison of size distributions of appressoria for mutant strains and the wild type. The sizes of appressoria were measured at 8 and 24 h postincubation. Dots within a violin plot indicate mean values. Different letters indicate a statistically significant difference among size distributions (*P < *0.05, LSD test, *n* > 100).

Despite deformation in spore morphology, conidia of the knockdown mutants were fully capable of germination (Fig. S4 at https://figshare.com/projects/MoRPD3/124153). However, appressorium formation on the hydrophobic surfaces of all the knockdown mutants was delayed to various degrees (Fig. 5A). In addition, examination of the diameter of appressoria at 8 h postincubation (hpi) showed that the mutant appressoria were significantly smaller than those of the wild-type strain and remained smaller even at 24 hpi, suggesting a potential defect in appressorium functionality (Fig. 5B and C). Conidia of the mutant were able to develop appressoria on a hydrophilic surface when cAMP was added, suggesting that *MoRPD3* functions in either a parallel pathway or upstream of the cAMP signaling pathway (Fig. S5 at https://figshare.com/projects/MoRPD3/124153). Collectively, these results indicate that *MoRPD3* does not contribute to the initiation of appressorium formation but to proper timing and morphogenesis.

### Dependency of rapamycin inhibition effect on MoRPD3.

**FIG 6:**
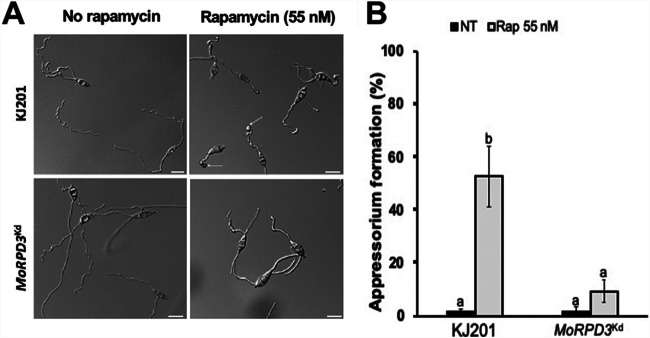
Dependence of rapamycin treatment effect on the *MoRPD3* mutant. (A) Micrograph showing appressorium formation on a noninductive (hydrophilic) surface with or without rapamycin. Triangles indicate incipient appressoria. (B) Quantitative analysis of appressorium formation on a noninductive surface with or without rapamycin (*n* > 100). Rap, rapamycin; NT, no treatment. Different letters on the bars indicate a statistically significant difference in mean values (*P < *0.05, LSD test, *n* > 100).

In S. cerevisiae, TOR is known to regulate the association of the Rpd3-Sin3 HDAC complex with ribosomal DNA (rDNA) chromatin. Therefore, ScRpd3 is required for the repression of ribosomal protein genes and 35S rDNA genes in response to the inhibition of TOR upon rapamycin treatment (22, 50). As rapamycin was shown in a previous study to induce the appressorium formation of *M. oryzae* conidia on hydrophilic surfaces (51), we tested whether such rapamycin-induced appressorium formation is dependent on MoRPD3. When rapamycin was added to germinating conidia of the wild type on a hydrophilic surface, a dramatic increase in the formation of incipient appressoria was observed. In stark contrast, the mutant conidia responded little to rapamycin treatment (Fig. 6A and B), indicating that rapamycin-induced appressorium formation on hydrophilic surfaces requires MoRPD3.

The observed dependency of rapamycin treatment’s effect on *MoRPD3* prompted us to examine the level of rRNA transcription from the wild-type and mutant strains with or without rapamycin for both mycelia and germinating conidia using qRT-PCR. As expected, treatment of the wild-type strain with rapamycin decreased the abundance of rRNA transcription in mycelial tissue, compared to that in the control (without rapamycin) (Fig. S6A at https://figshare.com/projects/MoRPD3/124153). Such a large reduction in the relative abundance of rRNA, however, was observed in the mycelia of *MoRPD3^kd^* mutants without rapamycin treatment (Fig. S6A), strongly suggesting a conserved role for MoRPD3 in the epigenetic regulation of rDNA loci in *M. oryzae* as well. For germinating conidia, qRT-PCR was carried out using RNAs extracted from germinating conidia at 2, 4, and 8 hpi on hydrophobic, hydrophilic, and hydrophilic surfaces with rapamycin treatment (Fig. S6B and C at https://figshare.com/projects/MoRPD3/124153). Two and 4 hpi represent the times when surface recognition and initiation of appressorium formation occur, respectively. A previous study showed that recognition of the hydrophobic surface results in the inactivation of TOR kinase (TOR_off_), which in turn leads to G_2_ cell cycle arrest and autophagy (45). Meanwhile, 8 hpi is the time for appressorium formation and maturation, which requires activated TOR kinase (TOR_on_)-mediated rRNA production. On the hydrophobic surface, the wild-type showed an increase in rRNA abundance over time, which is consistent with our expectation for TOR kinase activation and the subsequent production of rRNAs following recognition of surface hydrophobicity (Fig. S6B). Unlike with the wild-type, rRNA abundance did not increase significantly up to 8 hpi in the germinating conidia of the mutant. This may explain the delayed appressorium formation in the mutants. Interestingly, rRNA abundance remained low on hydrophilic surfaces for both the wild-type and mutant strains. Adding rapamycin to the germinating conidia on the hydrophilic surfaces caused little change in rRNA abundance. This pattern of rRNA abundance in germinating conidia highly correlated with the transcript abundance of *MoRPD3* (Fig. S6C at https://figshare.com/projects/MoRPD3/124153), suggesting that MoRPD3 is involved in the rRNA production of germinating conidia in a surface-dependent manner. However, rRNA abundance alone could not explain the dependency of rapamycin treatment’s effect on *MoRPD3.*

### Pathogenicity of MoRPD3*^kd^*.

**FIG 7:**
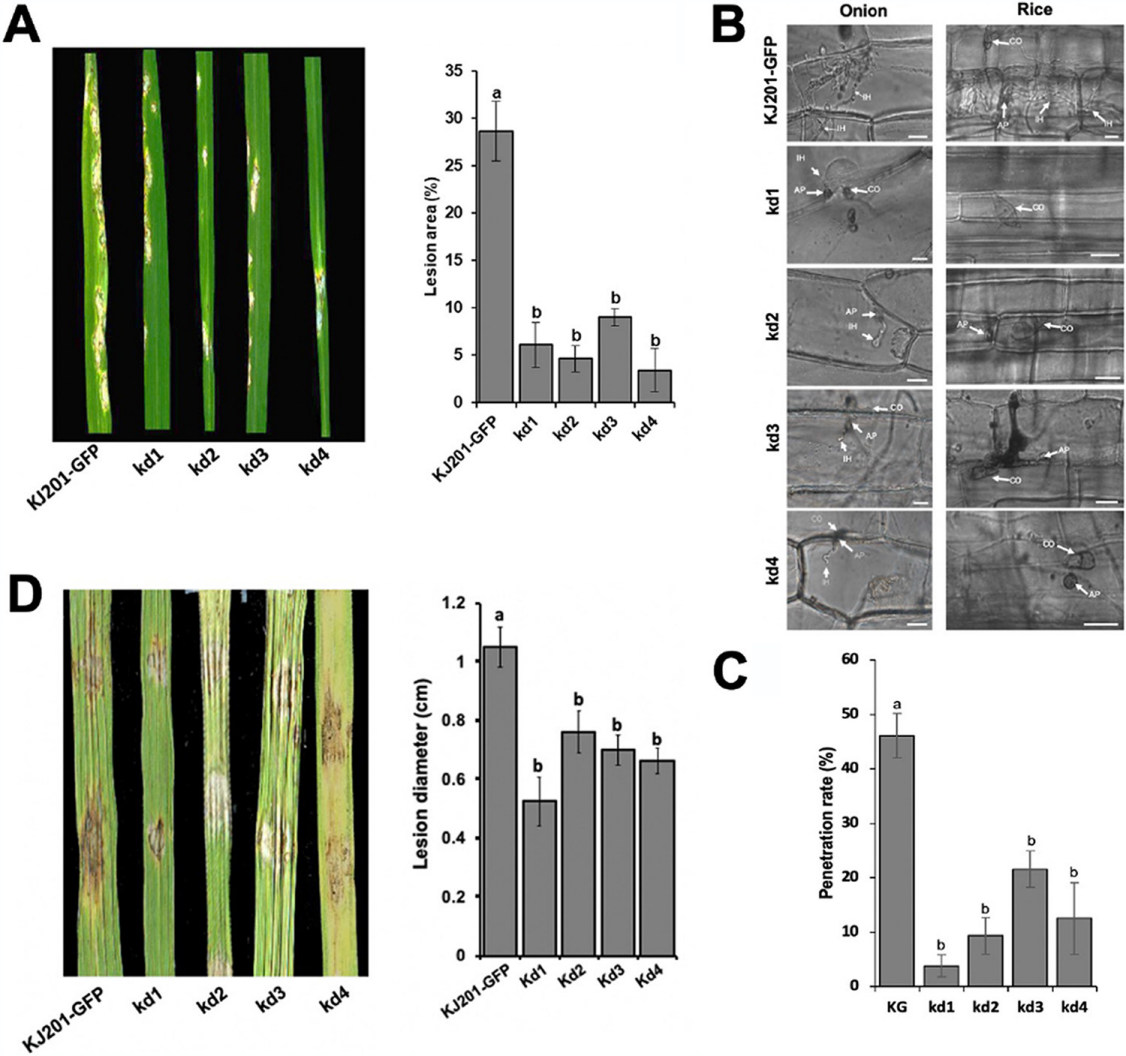
Requirement of *MoRPD3* for full virulence. (A) Result of the pathogenicity assay using the spray inoculation method. Images were taken 7 days postinoculation. (B) Onion epidermis and rice sheath for monitoring penetration and invasive growth. “CO,” “AP,” and “IH” indicate conidia, appressorium, and invasive hyphae, respectively. Scale bar, 20 μm. (C) Penetration rates of wild-type and knockdown strains. The number of appressoria that penetrated the rice cells was counted for each strain (at least 100 appressoria were observed). Error bars indicate standard errors. Different letters indicate a statistically significant difference in mean values (*P < *0.001, Tukey’s honestly significant difference [HSD] test). (D) Lesion development on rice leaves following wound inoculation. Images were taken 5 days postinoculation. Error bars indicate standard errors. Different letters indicate statistically significant differences in mean values (*P < *0.05, LSD test, *n* = 3).

In order to understand the role of *MoRPD3* during pathogenesis, conidia of mutants and the wild-type strain were harvested from OMA plates and then sprayed on the leaves of the susceptible rice cultivar Nakdongbyeo. When the development of disease lesions was evaluated 7 days postinoculation (dpi), leaves challenged with the wild-type strain exhibited numerous spindle-shaped lesions, whereas mutant-infected leaves showed a small number of tiny dark brown lesions and occasionally a few spindle-shaped lesions (Fig. 7A). Consistently with the visual inspection of lesion development, lesion areas on mutant-infected leaves were significantly smaller than those on the leaves inoculated with the wild type (Fig. 7A).

To delve into the reason(s) for the reduced virulence of *MoRPD3^kd^* strains, we monitored the appressorium-mediated penetration and invasive growth of the mutants using onion epidermis and rice sheath (Fig. 7B). In contrast to the wild-type strain, which was able to penetrate and grow inside the plant cells at 36 hpi, all the mutant strains showed either considerable delay or failure in appressorium-mediated penetration (Fig. 7B and C). Next, we tested whether the mutants were compromised in their ability to grow inside the plants using the wound inoculation method, through which the fungus was allowed to have direct access to the plant tissues without appressorium-mediated penetration. When spore suspensions were placed upon sites wounded using a pipette tip, the *MoRPD3^kd^* strains showed reduced lesion lengths, compared to those of the wild type, suggesting the role(s) of MoRPD3 during invasive growth (Fig. 6D). Consistently with the potential role of MoRPD3 in invasive growth, the MoRPD3-GFP signal was observed in invasive hyphae as well (Fig. S7A at https://figshare.com/projects/MoRPD3/124153). However, our assay using plate cultures supplemented with hydrogen peroxide suggested that such a reduction in invasive growth is unlikely to be due to the increased sensitivity of the mutants to reactive oxygen species produced by the host plant as a defense response (Fig. S7B at https://figshare.com/projects/MoRPD3/124153). All together, these results suggest that *MoRPD3* is required for appressorium functionality and growth inside the plant cells.

### MoRPD3 does not regulate TRK2 expression under low-K^+^ conditions.

The budding yeast possesses two genes, *Trk1* and *Trk2*, encoding the high- and low-affinity potassium ion transporters, respectively. Deletion of the high-affinity transporter gene *Trk1* has been shown to limit the growth of yeast cells on media containing low levels of K^+^. The *Rpd3* gene of the yeast was initially identified as a suppressor of the *Trk2* gene, since *Rpd3* mutation allowed the Δ*trk1* mutant to regrow in low-K^+^-containing media by increasing the expression of the *Trk2* gene (16).

**FIG 8:**
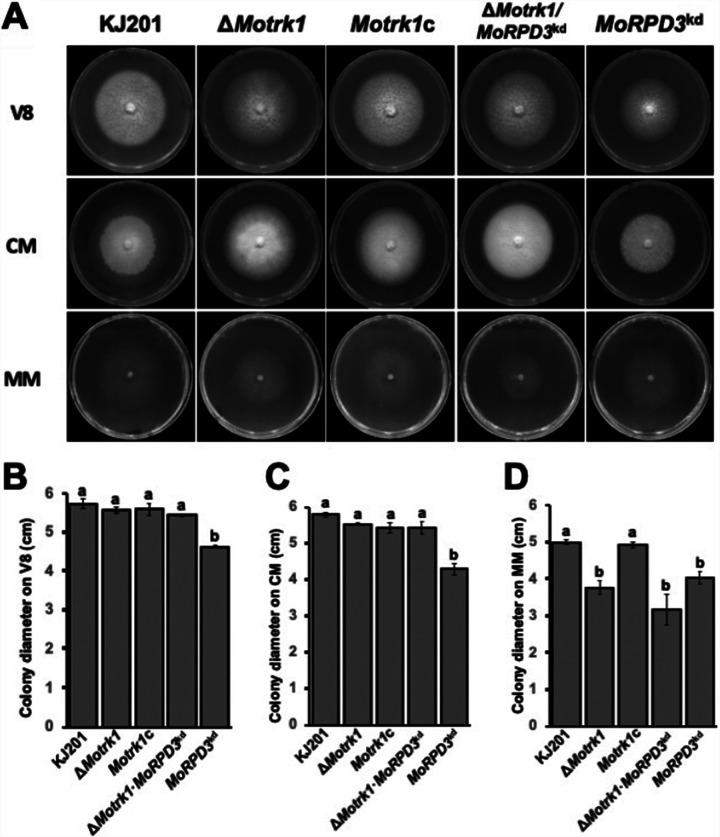
Genetic assay for testing the regulation of potassium transporter genes by the *MoRPD3* mutant. (A) Vegetative growth of the wild-type strain (KJ201) and the Δ*Motrk1*, *MoTRK1c* (Δ*Motrk1* complementation strain), Δ*Motrk1/MoRPD3^kd^*, and *MoRPD3^kd^* strains on V8 (B), complete medium (CM) (C), and minimal medium (MM) (D). MM contains low concentration of potassium ions. Different letters indicate statistically significant differences in mean values (*P < *0.05, LSD test, *n* = 3).

To test if such RPD3-mediated regulation of K^+^ transporters is conserved, we first searched the genome of *M. oryzae* for orthologs of Trk1 and Trk2. Our BLASTP search using the yeast Trk1 and Trk2 sequences revealed that there are four potential orthologs (MGG_06469 [51% identity], MGG_09119 [42% identity], MGG_07233 [34% identity], and MGG_04338 [32% identity]) in the fungal genome, as reported previously (52). Examination of protein domains showed that MGG_06469 and MGG_09119 possess a potassium transporter protein domain (IPR004473), while the remaining two did not. Considering their higher sequence identity and domain information, we designated MGG_06469 and MGG_09119 *MoTRK1* and *MoTRK2*, respectively. Deletion of the *MoTRK1* gene had a major impact on the production of asexual spores (Fig. S8A to C at https://figshare.com/projects/MoRPD3/124153), but it had no obvious effect on growth on V8 and complete media (0.5 g of KCl and 1.5 g of KH_2_PO_4_ per liter) or conidial germination and appressorium formation (Fig. 8A to C and see Fig. S8D and E at https://figshare.com/projects/MoRPD3/124153). However, the mutant showed an ∼28% reduction in growth on minimal medium containing a low concentration of potassium (0.2g of KH_2_PO_4_ per liter) (Fig. 8D). Such a decrease in growth and conidiation could be complemented by reintroduction of the *MoTRK1* gene into the deletion mutant. These results suggest the function of MoTRK1 as a bona fide potassium transporter.

Given the phenotypes of the Δ*Motrk1* strain, we hypothesized that if MoRPD3 negatively regulates *MoTRK2* transcription via histone deacetylation activity as in yeast, then depletion of *MoRPD3* in the Δ*Motrk1* strain should complement, at least partially, the growth defect of the mutant. To test this, we created a *MoTRK1* gene deletion in the *MoRPD3* silencing mutant background, allowing us to avoid tedious screening of the mutants for *MoRPD3* silencing. Silencing of *MoRPD3* in the Δ*Motrk1* strain, however, was not able to complement the growth defect of the mutant at all on media with low levels of K^+^, suggesting that a target gene of evolutionarily conserved HDAC is not conserved in *M. oryzae*.

## DISCUSSION

*RPD3* is a class I HDAC that has orthologs in diverse organisms, including prokaryotes (5). In mammals, RPD3 has been found to be associated with diseases such as cancer (8, 53). In *Drosophila*, it was shown to function in embryo segmentation (54). The plant RPD3 gene is involved in the regulation of growth and the development of plants (55–57). In the budding yeasts, Rpd3 has been shown to be involved in many cellular processes (5). In other yeast forms of fungi, such as C. neoformans and U. maydis, it was shown that *Rpd3* regulates development and pathogenesis (29, 30, 37).

Interestingly, unlike with the yeast form of fungi, deletion of *Rpd3* genes in some filamentous fungi seems to be lethal. For example, *RpdA* (Rpd3-type HDAC) genes in A. nidulans and A. fumigatus appear to be essential for growth and development (31, 32), although deletion of *Rpd3* did not cause lethality in other filamentous fungi, including Beauveria bassiana (*BbRpd3*), *Botrytis cinerea* (*BcRpd3*), and Fusarium graminearum (*FgRpd3*) (33, 34, 58). In our study, more than 1,000 transformants were screened in an attempt to isolate a *MoRPD3* gene deletion mutant, only to find that there were none. Such failure to obtain the deletion mutant is consistent with recently published data (35). This strongly suggests that deletion of *MoRPD3* is likely to be lethal. Initially, such lethality was attributed to the presence of a long C-terminal region of RPD3 in filamentous fungi, which is lacking in their yeast counterpart. However, nonlethal phenotypes of *RPD3* in *B.* bassiana, *B. cinerea*, and F. graminearum point to the degree of functional redundancy among class I HDACs and/or interacting partners of the C-terminal region as determining factors of lethality in different fungal species. Since the *M. oryzae* genome lacks genes encoding some orthologs among components of the yeast Rpd3 complex (see Table S1 at https://figshare.com/projects/MoRPD3/124153), *M. oryzae* may differ from S. cerevisiae and other filamentous fungi in some component(s) of the Rpd3 HDAC complex.

In this study, the MoRPD3-GFP fusion protein localized mainly to the nuclei of conidia, vegetative hyphae, and invasive hyphae, as expected for an HDAC, although faint GFP signals were also detected in the cytoplasm of conidia. The observed pattern of MoRPD3-GFP expression and localization suggests that (i) MoRPD3 is implicated in pathogenic development and invasive growth and (ii) it may have nonhistone target proteins in the cytoplasm. To date, none of the Rpd3-type HDACs have been reported to have nonhistone targets, although other members of the class I HDAC family have been shown to have deacetylase activity on nonnuclear proteins (28). Other observations showing the cytoplasmic localization of Rpd3 in A. nidulans (32), *B.* bassiana (58), and maize (*Zea mays*) (57) suggest, along with our data, the possibility of *Rpd3* of filamentous fungi having nonhistone targets, unlike with their yeast counterpart. Such a possibility needs to be addressed in future studies, as it may offer an explanation for species-specific phenotypes, including lethality, upon gene deletion. Unlike the *MoRPD3-GFP* construct, the *MoRPD3^Δ346–659^-GFP* construct was not able to fully complement the yeast *Rpd3* deletion mutant. This suggests that the C-terminal region of MoRPD3 is required either for the full functionality of MoRPD3 as an HDAC, probably through its contribution to the formation of an optimal three-dimensional structure, or for maintaining the stability of the protein. If the protein turnover rate is high enough to make futile our effort to detect GFP signals from strains carrying the *MoRPD3*^Δ^*^346–659^-GFP* construct, it is unlikely for the same construct to partially complement the yeast *Rpd3* deletion strain. Therefore, we conjecture that our failure to detect the GFP signal from strains harboring *MoRPD3*^Δ^*^346–659^-GFP* may be attributed primarily to inappropriate protein folding resulting from the lack of a C-terminal region, which we think might interfere with the folding of GFP sequences as well. These lines of data strongly suggest that unlike yeast, filamentous fungi, including *M. oryzae*, have evolved extended C-terminal regions that have an inextricable relationship with HDAC domain function. We also conjecture that the C-terminal domain of MoRPD3 might serve as a platform for interaction with other proteins, which would allow more spatial and temporal control of MoRPD3’s activity. The roles of the C-terminal regions warrant further investigation in the future.

Due to the lethality of *MoRPD3* deletion, alternatively, we generated knockdown mutants using the dual-promoter vector pSD1, which has been successfully applied to the silencing of genes in *M. oryzae* (49). Interestingly, our immunoblotting assay for H3K9ac, H3K18ac, and H4K5ac, which are some of the known substrates of Rpd3 in yeast, detected significant but small increases in acetylation levels from the silencing mutants, compared to those in the wild type. Lin et al. also performed immunoblotting experiments to detect changes in the overall acetylation levels of H3 histone proteins using anti-H3AcK (35). Their work showed quite a large decrease in the H3 acetylation level from that of a strain overexpressing *MoRPD3*. It is not possible to make a direct comparison with our data, since Lin et al. did not perform a quantitative analysis. We conjecture that these two factors may contribute to the marginal increase in histone acetylation levels from the silencing mutants. First, it is possible that depletion of MoRPD3 proteins in the silencing mutant has a positive effect on the regulation of histone acetylation. Second, *MoRPD3* expression in the overexpression strain was 12- to 15-fold higher than that of the wild type, while the transcript abundance of *MoRPD3* in the silencing mutants was approximately 2.5- to 5-fold lower than that in the wild type.

Silencing *MoRPD3* had little effect on the vegetative growth of the fungus but had a major impact on asexual sporulation, both in quantity and morphology. The role(s) of *Rpd3* orthologs in asexual reproduction in filamentous fungi has been poorly studied, probably owing to the severe growth defect of the deletion mutant, which does not allow further investigation. The decrease in the number of conidia from *MoRPD3^kd^* suggests that *MoRPD3* plays important roles during conidiogenesis via MoRPD3-mediated direct and indirect regulation of conidiation-related genes. Our data showed that the silencing of *MoRPD3* leads to increased sexual reproduction, in contrast to what occurs with asexual reproduction, suggesting that it is a negative regulator of sexual reproduction. In S. cerevisiae, *U. maydis*, and F. graminearum, Rpd3 appears to be a positive regulator of sexual reproduction (17, 30, 33). Field isolates of *M. oryzae* generally exhibit poor fertility, unlike the highly fertile F. graminearum and *U. maydis*, suggesting that this functional difference in *Rpd3* genes during sexual reproduction among different fungal species may be a key to elucidating the underlying differences in the levels of fertility of fungal species. However, more data should be forthcoming. Additionally, it should be noted that the degree of *MoRPD3* gene silencing during sexual reproduction might be affected by a sexual-stage-specific silencing mechanism, such as meiotic silencing by unpaired DNA (MSUD) (59), although little is known about whether MSUD exists and operates in *M. oryzae*.

Conidia of knockdown mutants were able to germinate and develop, with a little delay in time, smaller sizes of appressoria than those of the wild type. Such a delay and size decrease in appressorium formation are reminiscent of *cpka* mutants (60). However, it is not clear whether MoRPD3 is entwined with the cAMP signaling pathway in any way, although our experiment with cAMP on a hydrophilic surface does not exclude the possibility that MoRPD3 is upstream of the cAMP signaling pathway. In S. cerevisiae, rRNA loci and ribosomal protein (RP) genes are regulated by TOR, of which the inhibition with rapamycin releases Esa1 histone acetyltransferase from and simultaneously recruits the Rpd3-Sin3 complex to the promoters of rRNA and RP genes (21, 22). Recently, Sun et al. showed that appressorium formation requires temporary arrest of the cell cycle at the G_2_ checkpoint through inactivation of TOR signaling (45). Here, we demonstrated that rapamycin-induced appressorium formation on a hydrophilic surface is dependent on MoRPD3 and that MoRPD3 is required for the repression of rDNA loci upon rapamycin treatment. Based on our own data and what is known about yeast, it stands to reason that *MoRPD3* is involved in appressorium formation and morphogenesis via its regulation of ribosome biogenesis, which is inseparably intertwined with cell cycle control. As rRNA loci are located in nucleoli, the roles of MoRPD3 in regulating rRNA gene transcription and nucleolar structure in this filamentous fungus warrant further study. However, rRNA abundance alone could not explain the dependency of appressorium formation on hydrophilic surfaces by rapamycin treatment of MoRPD3. Therefore, it is tempting to conjecture that MoRPD3 is involved in the regulation of target genes in addition to the rDNA loci that are required for appressorium morphogenesis. Such a possibility should be explored in future studies.

Our pathogenicity assay using the spray inoculation method showed a significant reduction in the virulence of the mutant strains. Wound inoculation also showed considerable reduction in lesion length, suggesting that *MoRPD3* is required for invasive growth. Because the mutant strains were as resistant to hydrogen peroxide as the wild-type strain, we could rule out the possibility that the mutants are either defective in maintaining cell wall integrity or incapable of dealing with plant-derived reactive oxygen species. The strong GFP signal from the MoRPD3-GFP fusion protein during invasive growth suggests the possibility that MoRPD3-mediated histone deacetylation is involved in the regulation of chromatin stability and/or expression of genes encoding effectors. One thing that is rather counterintuitive and therefore worth noting here is that both our silencing mutant and the overexpression mutant reported by Lin et al. (35) exhibit similar defects in pathogenicity. We conjecture that the development of functional appressoria and invasive growth require the fungus to strike a subtle balance in *MoRPD3* expression, since these processes demand sophisticated and timely coordination between rRNA production (and, consequently, ribosome biogenesis) and the transcription of a specific set of developmental genes. The delicate nature of MoRPD3’s function during pathogenesis might explain at least in part why both the depletion and an excess of *MoRPD3* transcripts can lead to similar or the same phenotypic outcomes.

In summary, our data indicate that MoRPD3 is a conserved regulator responsible for the regulation of reproduction and pathogenic development throughout the life cycle of the rice blast fungus. Our experimental results suggest that MoRPD3-mediated regulation of rRNA transcription in a TOR-dependent manner may underlie not all but many of the phenotypes observed in the mutant. Further elucidation of the target genes and the potential regulation of nucleolar functions by MoRPD3 would provide insights into the evolution and implications of epigenetic mechanisms in fungal pathogenesis.

## MATERIALS AND METHODS

### Fungal isolates and culture conditions.

Wild-type strain KJ201 and the RFP-fused histone H1 (*H1-RFP*) transgenic strain were obtained from the Center for Fungal Genetic Resources (CFGR), South Korea. All strains, including mutants, were grown on oatmeal agar (OMA; 5% oatmeal [wt/vol] and 2% agar [wt/vol]) under continuous-lighting conditions and aeration to enhance sporulation or on V8 agar at 25°C for routine experimental work. For DNA and RNA isolation, 3- to 4-day-fresh hyphae were obtained from the complete medium broth (CM; 0.6% [wt/vol] yeast extract, 0.6% [wt/vol] Casamino Acids, and 1% [wt/vol] sucrose at 25°C with 120-rpm shaking). Selection of transformants was done using TB3 agar plates (0.3% [wt/vol] yeast extract, 0.3% [wt/vol] Casamino Acids, 1% [wt/vol] glucose, 20% [wt/vol] sucrose, and 0.8% [wt/vol] agar powder) supplemented with 200 ppm of hygromycin B or 400 ppm of Geneticin.

### Yeast strains, culture conditions, and transformation.

Saccharomyces cerevisiae strains, which were ATCC 201389 and ATCC 4011114, were obtained from the American Type Culture Collection (ATCC). All strains were grown in yeast extract-peptone-dextrose (YPD) growth medium (1% [wt/vol] yeast extract, 2% [wt/vol] peptone, and 2% [wt/vol] glucose) and YPD agar (YPD and 2% [wt/vol] agar) with or without 1.2 M NaCl at 30°C. Constructs containing full-length *MoRPD3* or *MoRPD3*-ΔC (MoRPD3 with C-terminal sequences removed) were cloned into the pYES2 vector. Yeast transformation was carried out using a Frozen-EZ yeast transformation II kit according to the manufacturer’s instruction. Transformants growing on synthetic dextrose (SD) minimal medium (0.67% [wt/vol] yeast nitrogen base without amino acids, 2% [wt/vol] glucose, and 2% [wt/vol] agar) containing 0.192% (wt/vol) yeast synthetic dropout medium supplements without uracil at 30°C.

### Vegetative growth, reproduction, and appressorium formation.

For measurement of radial growth, conidium production, conidial germination, and appressorium formation, strains were grown on OMA, CMA, and V8, for 9 days, in three replicates, under constant light to promote conidium production. Conidia were harvested from 9-day-old colonies using 5 ml of sterilized, distilled water and counted using a hemocytometer under a light microscope. The average size of conidia was estimated by measuring the lengths and widths of at least 100 conidia. Then, the number of septa was examined by observing septa following calcofluor white staining for at least 100 conidia per replicate. To monitor germination and appressorium formation, the concentration of conidial suspensions was adjusted to ∼5 × 10^4^ conidia/ml. A 40-μl suspension was placed on a hydrophobic surface (coverslips) and then incubated in a humidity chamber at 25°C for 24 h. The germination rate was calculated by counting the number of germinating spores out of at least 100 spores at 4 h postincubation (hpi). The appressorium formation rate was calculated by counting the number of appressorium-forming conidia out of at least 100 germinating conidia 8 to 24 hpi. The size of developed appressoria was estimated by measuring the lengths of at least 100 appressoria. All the assays were conducted with three technical replicates in three independent experiments. Sexual reproduction was observed by crossing the 4091-5-8 strain with the wild-type or mutant strain on oatmeal medium under an inductive condition. Perithecia were harvested from the plates, observed under a Leica DM2500 light microscope, and pictured with a Leica DFC7000 T digital camera using Leica Application Suite v4.

### cAMP and rapamycin treatment experiments.

Conidia were collected from 9-day-old cultures grown on oatmeal agar plates and then resuspended in sterilized distilled water to 5 × 10^4^ conidia/ml. Two hundred microliters of the spore suspension was dropped on a hydrophilic surface (glass slides) and then incubated in a humidity chamber at 25°C for 24 h to monitor appressorium formation. Rapamycin (55 μM) and cAMP (10 mM) were used to supplement conidial suspensions separately or in combination to test their effects on appressorium formation. The appressorium formation rate was calculated by counting at least 100 conidia per replicate. Slides were examined under a Leica DM2500 microscope and imaged with a Leica DFC7000 T digital camera using LAS X software.

### Pathogenicity assay.

For a pathogenicity test, a conidium suspension was either sprayed (∼5 × 10^4^ conidia/ml) on rice seedlings or directly placed onto wounded sites (∼2 × 10^5^ conidia/ml). Inoculated plants were incubated in a humidity chamber without light for 24 h and then transferred to an incubator for growth at 25°C with a 16-h photoperiod of fluorescent light. After 7 days postinoculation, lesion development was assessed and photographed. Plants grown in three pots were used for a pathogenicity assay of individual strains in each of three independent experiments.

### Generation of knockdown, knockout, and complementation strains.

Gene knockout constructs were prepared by double-joint PCR as previously described (61). For this, approximately 1.5 kb of flanking sequences of a target gene was amplified and fused to either side of the hygromycin resistance gene (HPH) cassette. Next, the resulting constructs were transformed into the wild-type protoplasts to generate transformants as previously described (62). Due to the difficulty of obtaining *MoRPD3* deletion mutants, knockdown mutants were generated as described previously (49). In summary, short exon sequences of *MoRPD3* were PCR amplified and inserted into the multicloning site of the dual-promoter vector pSD1. The resulting vector was used for the transformation of the wild-type strain expressing GFP. Transformants growing on TB3 medium containing hygromycin were subjected to PCR-based screening using specific primer pairs. Complementation of the *MoTRK1* deletion mutant was carried out by amplifying target gene and flanking sequences (∼1 kb on each side) from the wild type and introducing the resulting PCR products along with the pII99 plasmid harboring the Geneticin resistance gene into the mutant protoplasts. Transformants growing on gene-containing TB3 medium were selected and screened by PCR for the presence of the gene copy. Primers used in the generation of knockout and knockdown strains are listed in Table S2 at https://figshare.com/projects/MoRPD3/124153.

### Generation of the MoRPD3-GFP strain and fluorescence microscopy.

To observe the localization of MoRPD3, a translational fusion construct of MoRPD3 and GFP (expressed under the native promoter of the *MoRPD3* gene) was prepared and introduced into the wild-type strain and a strain expressing histone H1-RFP. For observation of the fluorescence signal in hyphae, a sterilized glass slide was immersed and coated with complete medium agar and then inoculated with mycelia of the strain expressing MoRPD3-GFP. Following incubation at 25°C for 5 days, nuclei were stained using the Hoechst 33342 dye (Invitrogen, Waltham, MA, USA) according to the manufacturer’s instructions prior to observation of MoRPD3-GFP localization in mycelia. In spores, the MoRPD3-GFP/H1-RFP strain was used to examine the localization of MoRPD3, compared to its localization in fungal nuclei. Microscopy was performed with a Leica DM2500 light microscope, and images were taken with a Leica DFC7000 T digital camera. Excitations were 340 to 380 nm for Hoechst 33342 dye, 480/40 nm for GFP, and 515 to 560 nm for RFP. Images were processed using LAS X software.

### RNA isolation and real-time PCR analysis.

RNA was extracted from 3- or 4-day-old fresh mycelia collected from complete medium broth cultures using an easy-spin total RNA extraction kit (iNtRON Biotechnology, Seoul, South Korea). cDNA was synthesized from 1 μg of total RNA using the ImProm-II reverse transcription system (Promega, Madison, WI, USA). Real-time PCR was conducted in a total reaction volume of 20 μl consisting of 2 μl of cDNA, 1 μM primer mix (forward and reverse), and 10 μl of 2× Power SYBR green PCR master mix (Applied Biosystems, Warrington, UK). The reaction conditions, set on the Applied Biosystems 7500 real-time PCR system (Applied Biosystems, Foster City, CA, USA) were 40 cycles of 15 s at 95°C, 30 s at 60°C, and 30 s at 72°C, with three technical replicates. Results of threshold cycles (*C_T_*) were averaged and then normalized as previously described (63). qRT-PCR experiments were conducted with three technical replicates in each of three biological replicates. Primers used in qRT-PCR are listed in Table S2 at https://figshare.com/projects/MoRPD3/124153.

### Western blot experiment.

Histone proteins were prepared from 3- or 4-day-old fresh mycelia collected from complete medium broth cultures using an EpiQuik total histone extraction kit (EpiGentek, Farmingdale, NY, USA). The histone proteins were separated on 12% SDS-polyacrylamide gels and transferred to an Immobilon-P PVDF (polyvinylidene difluoride) membrane (Millipore, Burlington, MA, USA). The blotted membrane was blocked with 5% skim milk in TBST (Tris-buffered saline, 0.1% Tween 20) for 16 h at 4°C. After being blocked, the membrane was incubated with primary antibody in TBST containing 5% skim milk for 1 h at 25°C; following incubation, the membrane was washed with TBST three times for 10 min. After being washed, the membrane was incubated with secondary antibody in TBST containing 5% skim milk for 1 h at 25°C; following incubation, the membrane was washed with TBST three times for 10 min. The antigen-antibody complexes were detected using WESTR ETC C ULTRA 2.0 (Cyanagen, Bologna, Italy), and images were acquired and analyzed using FUSION Solo-X (Vilber, Marne-la-Vallée, France). The antibodies and their dilutions were as follows: anti-H3K9ac (ab4441, 1:1,000 dilution; Abcam, Cambridge, UK), anti-H3K18ac (ab1191, 1:1,000 dilution; Abcam, Cambridge, UK), anti-H3 (ab1791, 1:1,000 dilution; Abcam, Cambridge, UK), anti-H4K5ac (ab51997, 1:1,000 dilution; Abcam, Cambridge, UK), anti-H4 (ab31830, 1:1,000 dilution; Abcam, Cambridge, UK), anti-rabbit IgG H&L (ab7090, 1:5,000 dilution Abcam, Cambridge, UK), and anti-mouse IgG H&L (ab6789, 1:5,000 dilution; Abcam, Cambridge, UK).
